# Oral Health Survey in Burundi; Evaluation of the Caries Experience in Schoolchildren Using the DMFT Index

**DOI:** 10.3390/medicina59091538

**Published:** 2023-08-25

**Authors:** Demetrio Lamloum, Marco Dettori, Pino La Corte, Maria Ruth Agnoli, Andrea Cappai, Arianna Viarchi, Antonella Arghittu, Thomas Gerhard Wolf, Paolo Castiglia, Guglielmo Campus

**Affiliations:** 1 Department of Public Health, Experimental and Forensic Medicine, University of Pavia, 27100 Pavia, Italy; demetrio.lamloum@students.unibe.ch; 2 Department of Restorative, Pediatric and Preventive Dentistry, University of Bern, 3012 Bern, Switzerland; madettori@uniss.it (M.D.); thomas.wolf@unibe.ch (T.G.W.); 3 Department of Medicine, Surgery and Pharmacy, University of Sassari, 07100 Sassari, Italy; aarghittu@uniss.it (A.A.); paolo.castiglia@uniss.it (P.C.); 4 Solidarietà Medico Odontoiatrica nel Mondo (SMOM) ODV, 20143 Milan, Italy; pinolacorte@fastwebnet.it; 5 Faculty of Dentistry, Universidad Alfonso X El Sabio, 28691 Madrid, Spain; magno@uax.es; 6 Department of Architecture, Design and Urban Planning, University of Sassari, 07041 Alghero, Italy; a.cappai7@phd.uniss.it; 7 Section of Odontostomatologic Surgery, University of Perugia, 06126 Perugia, Italy; ariannaviarchi@gmail.com; 8 Department of Periodontology and Operative Dentistry, University Medical Center of the Johannes Gutenberg-University Mainz, D-55131 Mainz, Germany; 9 Department of Cariology, Saveetha Dental College and Hospitals, Chennai 600077, India

**Keywords:** caries epidemiology, children, DMFT index, Burundi

## Abstract

*Background and objectives:* There are no data on oral health in the population of Burundi. This study aimed to describe the oral health status of schoolchildren in Burundi using the dmft/DMFT index for the first time. *Materials and methods:* The study was designed as a cross-sectional population-based epidemiological survey. The survey was designed according to the WHO methodology for oral health surveys. Oral examinations were conducted in school rooms using a dental mirror, probe, and headlight. The following characteristics of primary dentition status were recorded: decayed (d/D), missing (m/M), and filled (f/F) teeth, and the dmft/DMFT (d + m + f t/D + M + F T) index was calculated for each subject. Quantitative and qualitative variables were represented by measures of position and variability. One-way ANOVA was used to assess differences between parametric variables. Logistic regression was performed for total caries experience and gender, age groups, living area, and geographical provinces. *Results:* A total of 1902 children were examined, 1007 (52.94%) six-year-olds and 895 (47.06%) in the older group. The dmft/DMFT and subgroups were statistically significantly different in terms of age groups, living areas, and geographical regions (dmft/DMFT d-subgroup and D-subgroup *p* < 0.01), but only for DMFT for sex. The ORs estimated by logistic regression by total caries experience showed a protective effect for 12 year old subjects and those living in southern provinces, an OR of 0.52 (95%CI 0.43–0.64) and an OR of 0.26 (95%CI 0.21–0.32), respectively. *Conclusions:* Dental caries in African countries, including Burundi, remains a major problem affecting the general health and wellbeing of the population. Tackling untreated caries requires a multifaceted approach, including strengthening oral health infrastructure, promoting oral health education, providing affordable dental services, and encouraging healthier eating habits.

## 1. Introduction

In the African region, about half of the population suffers from oral diseases, most notably dental caries, periodontal disease, and tooth loss [1,2]. African countries face a disproportionate burden of tooth decay due to a number of factors, including limited access to dental care, poor oral hygiene practices, inadequate oral health education, and lack of preventive measures [3,4].

Caries prevalence has been declining in high-income countries (HICs) since the 1970s [5], but population data from many low-income countries (LICs) remain scarce. Management cost for oral diseases in LICs is skyrocketing [6]. Their impact affects quality of life, child growth, and daily activities [7,8]. The high rate of untreated caries in LICs is related to local endemic barriers, such as reduced human resources, lack of infrastructure, and poor information [9].

While numerous oral health studies have been conducted in Tanzania, Ghana, Ethiopia, Burkina Faso, and other African countries [10,11,12,13,14], there are limited studies on pediatric caries prevention and treatment in Burundi. The Republic of Burundi is a landlocked country in East Africa, situated in the African Great Lakes Region, to the east of the Democratic Republic of the Congo. Burundi consists of 18 regions, roughly divided into a northern hilly and mountainous part and a southern plain and with a population of 12.55 million. It has a GDP per capita of USD 221.48 [15]. According to the United Nations Development Program (UNDP), it is one of the least developed countries in the world in terms of life expectancy, literacy, and per capita income, ranking 187th out of 189th [16]. Despite the scarce economic resources and the fragmented public health system, oral health is included as a “public health problem” in the country’s national agenda and the national long-term health policy (Plan National de la Santé) [17], but several constraints limit the resilience of the local health system. There is a lack of oral health promotion and primary prevention of caries in Burundi [18,19], and the economic situation poses a challenge to individuals seeking dental care. The majority of the population faces financial barriers to accessing care. The risk of catastrophic health expenditure hinders regular dental check-ups, treatment, and preventive measures [20]. Strengthening the national oral healthcare systems seems pivotal [21]. Poor dental care infrastructure, particularly in underserved areas, lack of oral health education, and insufficient numbers of dental clinics, oral health professionals, and educational interventions need to be addressed [22,23,24,25,26]. LICs are faced with the challenge of the increasing caries prevalence, as the upward trend is mainly due to cariogenic dietary habits, poor oral hygiene, and fragmented care services. Data are scattered, and many countries have never had an epidemiologic survey [3]. Hence, the aim of this study was to present the first survey on oral health among schoolchildren in Burundi using the dmft/DMFT index. This will allow future national integrated strategies for the provision of primary healthcare and promote cost containment in health policy planning and development.

## 2. Materials and Methods

### 2.1. Study Design and Setting

**Figure 1:**
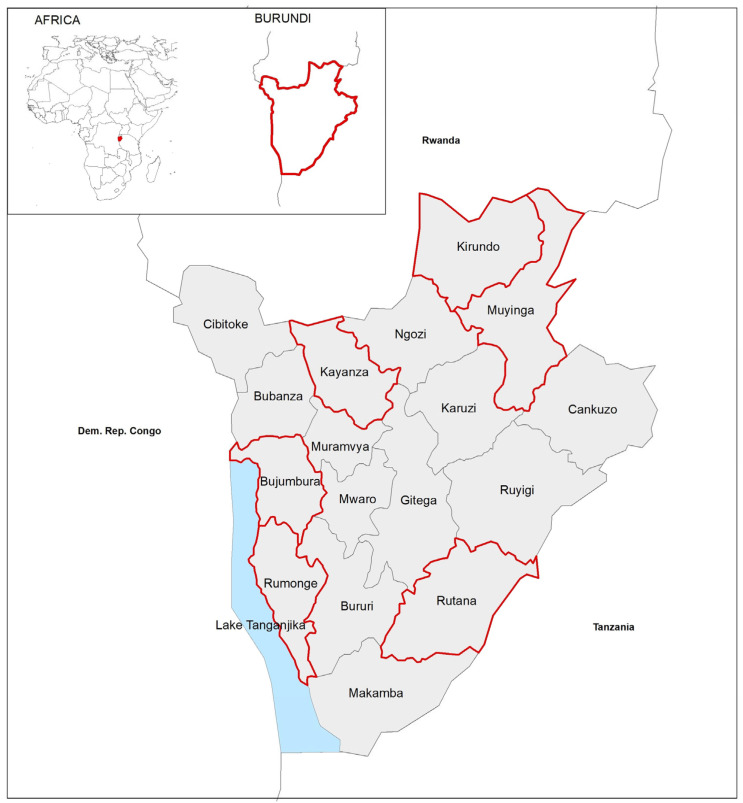
Geographical localization of Burundi, and areas of the study. The colored lines enclose the areas where the study took place.

The study was conducted in Burundi (Figure 1) and was designed as a cross-sectional population-based epidemiology survey. The University of Bern (Switzerland) and the University of Sassari (Italy) collaborated in the design and implementation of the study.

### 2.2. Methods

Sample size was assessed using the freeware online application openepi (http://www.openepi.com version 3, (accessed on 10 July 2023)), taking into account that no data were available; therefore, an expected prevalence of 50% was considered [27,28], as the expected prevalence was within the range of 10% and 90% of the prevalence. A confidence level of 97% was used. The number returned was 471; however, as Burundi could be divided into two main areas, the sample size was doubled. This strategy resulted in a sample that was self-weighting. Each child’s parents/caregivers received a leaflet explaining the aim of the study and requesting the child’s participation. Only children whose parents/caregivers signed the participation form were enrolled. All the subjects belonged to two age groups: 6 and 12 years old.

The survey method was carried out after a calibration process with caries lesions detected on images and clinically by four examiners. The calibration process was carried out first on images and then on 36 subjects equally distributed between the two age groups. Intra- and inter-observer reliability was assessed using Cohen’s kappa score.

The survey was designed according to the WHO methodology for oral health surveys [29]. Oral examinations were conducted in school rooms using a dental mirror, probe, and headlight. The following characteristics of the primary dentition status were recorded: decayed (d/D), missing (m/M), and filled (f/F) teeth, and the dmft/DMFT (d + m + f t/D + M + F T) index was then calculated for each subject.

### 2.3. Data Collection and Analysis

Data were entered into Excel (Microsoft Office, Microsoft Corporation, Redmond, WS, USA) and analyzed using STATA^®^ 17.0 statistical software (StatCorp., Austin, TX, USA) at a statistically significant level of *p* < 0.05. Total caries experience was converted as a dichotomous variable on the basis of the dmft/DMFT index (0 = caries free; 1 = at least one tooth with a history of caries regardless of whether the active lesion tooth was extracted or filled for caries). Subjects were grouped according to whether they lived in the northern (Kayanza, Muyinga, and Kirundo) or southern (Rumonge, Rutana, and Bujumbura) regions. The living area (urban or rural) was also taken into account. Qualitative variables were described in terms of absolute and relative frequencies. Associations between categorical variables were tested using Pearson’s chi-square. Quantitative variables were represented by measures of position and variability. One-way ANOVA was used to evaluate the differences between parametric variables. Multivariate analysis was performed using logistic regression for total caries experience and gender, age groups, living area, and geographical provinces. The presence of a possible effect modifier was assessed by cross-tabulation and generation of dummy variables tested by the above logistic regression analysis. The map of Burundi was retrieved from the World Atlas (https://www.worldatlas.com/maps/burundi, (assessed on 10 July 2023)) and ArcGIS software was used for geographic mapping and the shape file generation (version 10.8.2, Redlands, CA, USA).

## 3. Results

Intra- and inter-observer agreement, assessed using Cohen’s Kappa score, was good (0.78–0.84 and 0.73–0.89 ranges for Intra- and inter-observer agreement, respectively). A total of 1902 children were examined, 1007 (52.94%) in the six-year-old group and 895 (47.06%) in the older group (Table 1).

**Figure 2:**
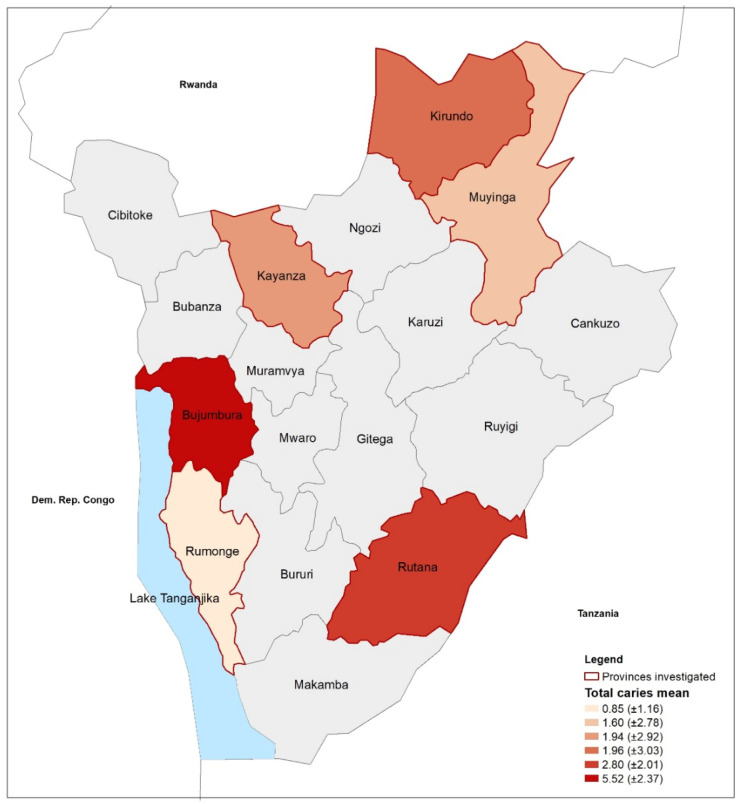
Total caries experience expressed as mean and standard deviation in the different areas of the study.

**Figure 3:**
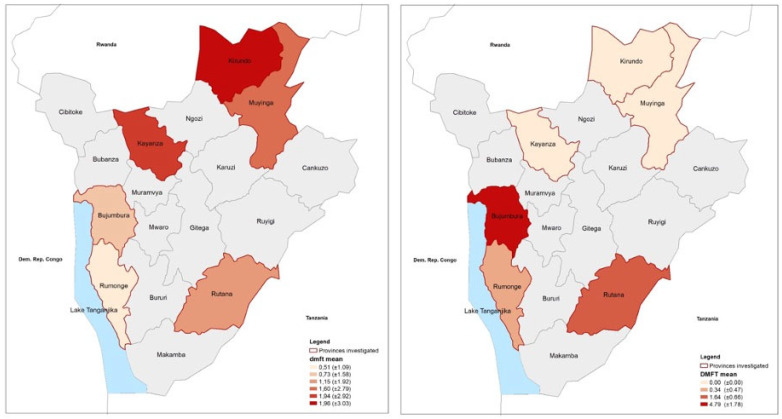
Caries experience in primary and permanent dentition dmft/DMFT, as the mean and standard deviation in the different areas of the study.

With regard to sex distribution, no statistically significant differences were observed with respect to age groups (*p* = 0.74), living area (*p* = 0.65), and regions (*p* = 0.17). Caries, dmft/DMFT, and subgroups, (Table 2, Figure 2) were statistically significantly different between age groups, living areas and geographical regions (dmft/DMFT d-subgroup and D-subgroup *p* < 0.01), but only for DMFT for sex (Figure 3).

The difference between filled teeth (f/F) was not computed as the number of fillings was too low. The ORs estimated by logistic regression (Table 3) by total caries experience showed a protective effect for the 12 year old subjects and those living in southern provinces an OR of 0.52 (95%CI 0.43–0.64) and an OR 0.26 (95%CI 0.21–0.32), respectively.

The association in the logistic regression showed an effect modifier of area of residence (urban or rural) with geographical gradient. Cross-tabulation between living area and geographical area (χ^2^ = 233.12 *p* < 0.01) showed an inversely proportional association. A living area/geographical regions dummy variable was created, which yielded a protective OR in bivariate regression (people living in urban areas in the south had a 50% lower risk of having caries).

## 4. Discussion

This study provides the first data on the oral health status of schoolchildren in Burundi. A higher dmft at 6 years than DMFT at 12 years was noted. Previous studies from Ethiopia [30] and Tanzania [31] showed similar results.

The analysis showed a higher prevalence of caries in females. These data are consistent with trends in East Africa [3]. Due to premature eruption, female teeth are exposed earlier to the oral environment, bacteria, and bacterial substrates than males of the same age [32]. Dietary habits are crucial, and the exposure of females to food preparation is a direct reason, due to easier access to food and snacks outside mealtimes [32].

In the present survey, children aged 6 years were keener to develop caries in the primary dentition than in the permanent dentition at the age of 12 years. The prevalence of caries is considered age-dependent [3]. Inaccurate tooth brushing techniques are associated with a higher prevalence of caries. The highest plaque reduction was found in the 12-year-old age group and the lowest in the 6-year-olds, with no difference between the sexes [33].

Although urban populations generally report higher caries experience than their rural counterparts, a higher caries experience among children from rural areas and for the northern provinces of Kayanza, Muyinga, and Kirundo was observed. It is necessary to emphasize that the majority of the population lives in rural areas with lower per capita income. There are significant differences in the distribution of oral health services, accessibility, utilization, and outcomes between urban and rural areas in both HICs and LICs [34]. Daily consumption of processed foods and sugar is relatively higher in urban areas than in rural areas [35]. Urban populations appear to be more aware of the importance of positive dental behaviors, such as tooth cleaning and regular dental visits as oral disease prevention techniques. Rural populations also appear to be less informed about the role of fluoride in caries [36,37]. The results show that the D component is disproportionately high in all six regions. Similarly, caries management seems to be a neglected area. The F-component had a negligible contribution in our study, as almost no fillings were restored during the screening process [38,39,40]. Data are consistent with neighboring countries [3]. Several factors may be responsible for the high proportion of untreated caries and lack of caries treatment. The high costs associated with dental treatment and the lack of providers are important factors. Dental treatment can be costly for an average Burundian family. Implementing policies to ensure affordability and accessibility of dental services can reduce barriers to oral healthcare, as safety nets, insurance coverage, and public health initiatives are not reported [41,42].

Second, the availability and distribution of facilities and personnel represent a prerequisite for an efficient service delivery system and influence the type of services provided and the number and type of patients seen [43]. Dental shortages are often endemic on the African continent [44]. Information on the health infrastructure in Burundi is lacking, as is information on the number and location of staff. The WHO database counts 14 dentists in the country, 0.012 per 10,000 inhabitants (HICs ratio is 1:2000), according to latest data from 2018 [26]. Strengthening the dental care infrastructure through governments and international organizations will be a valid alternative impacting the caries burden, including promoting the role of mid-level providers such as dental therapists [45,46,47,48].

Parental education and income often determine access to care and preventive measures such as low-calorie, sugar-based diets, and daily use of toothpaste and toothbrushes [49]. Integrating oral health education into the national curriculum and community outreach programs can increase awareness of oral hygiene practices, the importance of regular dental check-ups, and caries prevention. Cost-effective practices at the primary care level can also have long-term results, such as sodium fluoride mouthwash for primary school children (6–12 years) and providing varnish therapy twice a year for primary school children (6–14 years).

Many African countries face several challenges in promoting healthy diets and reducing the intake of cariogenic substances [50]. In addition to poverty-related inaccessibility to healthy diets, differences in fruit and vegetable intake can be attributed to low awareness and knowledge. Although dietary intake in the Great Lakes Region is dominated by traditional local foods such as sweet potatoes, cassava, and maize, intake of so-called ‘weekend foods’ and Western foods (i.e., sugary drinks and snacks) is increasing rapidly [51].

At the local level, the dietary transition that has introduced soft drinks, fast foods, and Western brand names to the continent is a national phenomenon that is progressing rapidly, especially in urban areas [52]. By promoting healthy eating habits, local authorities can raise awareness of their impact on oral health and noncommunicable diseases in general, taking into account public health campaigns and regulations.

This study had some limitations. The sample of schoolchildren considered was exclusively from public schools. Public schools are publicly funded and have subsidized tuition fees, whereas private schools are privately funded and have high tuition fees. Although public schools represent the vast majority of the population, different risk factors may be associated with the more affluent individuals attending private schools. In addition, data on covariates were not collected during the screening. Information on diet, oral hygiene habits, income, and family education level is missing.

## 5. Conclusions

Dental caries in African countries, including Burundi, remains a major problem affecting the general health and well-being of the population. As shown by this study, untreated caries prevalence is sky-high, and caries treatments are completely absent. Providing effective measures seems a priority, and multifaceted comprehensive approaches can be a solution. By prioritizing oral health and untreated caries as part of a broader health agenda, Burundi can work toward reducing its health and social impact, particularly in more neglected areas.

## Figures and Tables

**Table 1:** Demographic characteristics (sex, age groups, living area, provinces) of the enrolled sample.

	Sex		
	Males n (%)	Females n (%)	Total n (%)
6-year-olds	476 (47.27)	531 (52.73)	1007 (52.94)
12-year-olds	430 (48.04)	465 (51.96)	895 (47.06)
	Pearson χ^2^_(1)_ = 0.11 *p* = 0.74		
Urban	286 (46.89)	324 (53.11)	610 (32.07)
Rural	620 (47.99)	672 (52.01)	1292 (67.93)
	Pearson χ^2^_(1)_ = 0.20 *p* = 0.65		
Kayanza	125 (41.39)	177 (58.61)	302 (15.88)
Muyinga	247 (49.30)	254 (50.70)	501 (26.34)
Kirundo	152 (50.84)	147 (49.16)	299 (15.72)
Northern provinces	524 (47.55)	578 (52.45)	1102 (57.94)
Rumonge	149 (49.67)	151 (50.33)	300 (15.77)
Rutana	136 (45.33)	164 (54.67)	300 (15.77)
Bujumbura	97 (48.50)	103 (51.50)	200 (10.52)
Southern provinces	382 (47.75)	418 (52.25)	800 (42.06)
	Pearson χ^2^_(5)_ = 7.70 *p* = 0.17		

**Table 2:** Caries diseases indices (dmft/DMFT) sorted by sex, age groups, living area, and provinces of the enrolled sample.

(**A**) **Primary Dentition**				(**B**) **Permanent Dentition**				
	Males mean ± SD (range)	Females mean ± SD (range)	One-way ANOVA *p*-value	Sex		Males mean ± SD (range)	Females mean ± SD (range)	One-way ANOVA *p*-value
d	1.25 ± 2.33 (0–13)	1.33 ± 2.37 (0–16)	0.51		D	0.71 ± 1.46 (0–9)	0.89 ± 1.74 (0–12)	0.01
m	0.1 ± 0.43 (0–4)	0.09 ± 0.48 (0–6)	0.70		M	0.01 ± 0.13 (0–2)	0.01 ± 0.12 (0–2)	0.71
f	0.01 ± 0.17 (0–5)	0.00 ± 0.07 (0–2)	0.66		F	--	0.00 ± 0.03 (0–1)	--
dmft	1.35 ± 2.47 (0–13)	1.42 ± 2.50 (0–16)	0.55		DMFT	0.72 ± 1.46 (0–9)	0.90 ± 1.75 (0–12)	0.02
	6-year-olds mean ± SD (range)	12-year-olds mean ± SD (range)	One-way ANOVA *p*-value	Age groups		6-year-olds mean ± SD (range)	12-year-olds mean ± SD (range)	One-way ANOVA *p*-value
d	2.28 ± 2.83 (0–16)	0.18 ± 0.67 (0–7)	<0.01		D	0.28 ± 0.77 (0–6)	1.39 ± 2.05 (0–12)	<0.01
m	0.17 ± 0.59) (0–6)	0.01 ± 0.17 (0–3)	<0.01		M	0.01 ± 0.10 (0–2)	0.02 ± 0.15 (0–2)	0.25
f	0.01 ± 0.17 (0–5)	--	--		F	--	0.00 ± 0.03 (0–1)	--
dmft	2.45 ± 2.97 (0–16)	0.19 ± 0.70 (0–7)	<0.01		DMFT	0.29 ± 0.78 (0–6)	1.41 ± 2.06 (0–12)	<0.01
	Urban mean ± SD (range)	Rural mean ± SD (range)	One-way ANOVA *p*-value	Living Area		Urban mean ± SD (range)	Rural mean ± SD (range)	One-way ANOVA *p*-value
d	1.03 ± 1.86 (0–11)	1.41 ± 2.55 (0–16)	<0.01		D	0.35 ± 0.91 (0–7)	1.02 ± 1.82 (0–12)	<0.01
m	0.10 ± 0.51 (0–6)	0.09 ± 0.42 (0–4)	0.59		M	0.01 ± 0.09 (0–1)	0.01 ± 0.14 (0–2)	0.36
f	0.01 ± 0.22 (0–5)	--	--		F	0.00 ± 0.04 (0–1)	--	--
dmft	1.13 ± 2.02 (0–12)	1.50 ± 2.68 (0–16)	<0.01		DMFT	0.36 ± 0.92 (0–7)	1.03 ± 1.82 (0–12)	<0.01
	Northern regions mean ± SD (range)	Southern regions mean ± SD (range)	One-way ANOVA *p*-value	Geographical regions		Northern regions mean ± SD (range)	Southern regions mean ± SD (range)	One-way ANOVA *p*-value
d	1.70 ± 2.77 (0–16)	0.73 ± 1.45 (0–10)	<0.01		D	1.17 ± 1.94 (0–12)	0.30 ± 0.75 (0–6)	<0.01
m	0.10 ± 0.48 (0–6)	0.09 ± 0.42 (0–4)	0.67		M	0.00 ± 0.05 (0–1)	0.03 ± 0.19 (0–2)	<0.01
f	--	0.01 ± 0.19 (0–5)			F	--	0.00 ± 0.04 (0–1)	--
dmft	1.80 ± 2.89 (0–16)	0.81 ± 1.60 (0–11)	<0.01		DMFT	1.17 ± 1.94 (0–12)	0.32 ± 0.80 (0–6)	<0.01

**Table 3:** Logistic estimates of the model for total caries experience and gender, age groups, living area, and geographical region.

Total Caries Experience	Bivariate Analysis		Multivariate Analysis	
	OR (95% CI)	*p*-Value	OR (95% CI)	*p*-Value
Sex (*females*)	1.19 (1.00–1.42)	0.07	1.20 (1.00–1.47)	0.06
Age groups (*12-year-olds*)	0.62 (0.52–0.75)	<0.01	0.52 (0.43–0.64)	<0.01
Living area (*Rural*)	1.76 (1.45–2.14)	<0.01	1.20 (0.96–1.49)	0.104
Geographical regions (*Southern*)	0.26 (0.22–0.32)	<0.01	0.26 (0.21–0.32)	<0.01
Living area/geographical provinces (*Rural*/*Southern*)	0.48 (0.43–0.55)	<0.01		

## Data Availability

Data are available on request.
